# Good Practice for Treatment-Resistant Depression during SARS CoV – 2 outbreak: are ketamine infusions an effective alternative for TRD patients? A case series

**DOI:** 10.1192/j.eurpsy.2023.1779

**Published:** 2023-07-19

**Authors:** N. Ragone, B. Barbini, G. Perrozzi, C. Colombo

**Affiliations:** San Raffaele Ville Turro Hospital, Milano

## Abstract

**Introduction:**

The Mood Disorder ward, in San Raffaele Turro Hospital, is one of the reference centers for the cure of Treatment-Resistant Depression (TRD), mainly due to the use of Electroconvulsive Therapy (ECT). During the pandemic period, in particular, in 2020, such a procedure was discontinued because it is considered aerosolizing. For this reason, we enhanced already available treatments for TRD; among those one of the most effective is the use of endovenous (EV) ketamine. It’s been more than 20 years since the first time a double-blind randomized placebo-controlled study demonstrated the rapid antidepressant effects of endovenous (EV) ketamine after a single dose (0.5 mg/kg infused in 40 minutes) in 7 patients. Ketamine, an anesthetic drug, has also analgesic, anti-inflammatory, and antidepressant properties. These effects are mainly due to non-competitive antagonism on the NMDA receptor (N-methyl-D-aspartate). We introduce our clinical experience in 7 cases of treatment-resistant depressed (TRD) inpatients; all of them show a high level of pharmacoresistance, assessed in the third degree of Thase Stages (2 or more SSRI/SNRI + at least 1 TCA); 3 of them were previously treated with a complete cycle of Electroconvulsive Therapy (ECT).

**Objectives:**

Assess the efficacy and tolerability of EV ketamine with particular regard to patients previously treated with ECT.

**Methods:**

7 TRD patients (4 females; 3 males) were recruited in San Raffaele Turro Hospital in April 2020. All patients (6 unipolar and 1 bipolar) were diagnosed with a Major Depressive Episode according to DSM-5 criteria. We administered, under anesthesiological supervision, EV ketamine, 0.5 mg/kg in 40 minutes, twice a week, for three weeks. Every morning medication was postponed on the days of infusion. Clinical scales (HAM-D, SSI, HAMD-A; MADRS, CADSS) were administered to assess symptoms and side effects before, during, and after every administration. Moreover, clinical efficacy’s been assessed in 2 follow-ups: at 3 and 6 months.

**Results:**

4 patients were in remission (final HAM-D score <8) at the end of the treatment. 4 patients confirmed clinical response (final HAM-D score < 50 % respect baseline value) at the first follow-up. 4 Out of 7 patients were in complete remission at 6 months, and just one of them was between those remitted at the end of the treatment. 4 Out of 4 patients were in complete remission at six months follow-up; 3 of them underwent a cycle of ECT during the course of their illness.

**Conclusions:**

The use of EV ketamine in our TRD patients showed good effectiveness and tolerability. Data on long-term effectiveness are promising, a previous ECT seems to be a predicting factor of remission at follow-up, but not of the end-treatment response. Given that, future research is needed in order to identify predicting factors on relapse prevention efficacy.

**Disclosure of Interest:**

None Declared

